# Higher orthorexia tendency among female fashion models: an empirical international study

**DOI:** 10.1007/s40519-024-01674-4

**Published:** 2024-06-27

**Authors:** Nikolett Bogár, Szilvia Dukay-Szabó, Dávid Simon, Ferenc Túry

**Affiliations:** 1 Faculty of Medicine, Institute of Behavioural Sciences, Semmelweis University, Üllői út 26, Budapest, 1085 Hungary; 2 ELTE Eötvös Loránd University, Budapest, Hungary; 3 Department of Statistics, Faculty of Social Sciences, Eötvös Loránd University, Budapest, Hungary

**Keywords:** Orthorexia nervosa, Healthy eating, Fashion models, Thin beauty ideal, Obsession, Eating disorder

## Abstract

**Purpose:**

Female fashion models are more at risk for developing eating disorders than non-models due to the intense occupational pressure they face. The present study focuses on assessing whether female models are more prone to report orthorexia nervosa signs and symptoms than non-models.

**Methods:**

Female fashion models (*n* = 179, mean age: 25.9 SD = 4.40 years) and an age adjusted control group (*n* = 261, mean age: 25.0 SD = 4.97 years) were selected by snowball sampling. Participants filled out an online survey containing anthropometric questions and the 18-item Eating Habits Questionnaire.

**Results:**

According to BMI, fashion models were underweight (mean BMI = 18.1 SD = 1.68) while control participants’ BMI was in the normal range (mean = 22.1 SD = 4.23, *p* < 0.001). On all three of Eating Habits Questionnaire subscales fashion models showed significantly higher average value (Knowledge subscale: *M* = 2.42 among models versus *M* = 2.08 in the control group, *p* < 0.01, Cohen’s *d* = 0.52; Problems subscale: *M* = 1.93 among models versus *M* = 2.61 in the control group, *p* < 0.01, Cohen’s *d* = 0.49; Feelings subscale: *M* = 3.20 among models versus *M* = 2.96 in the control group, *p* < 0.01, Cohen’s *d* = 0.38). Orthorexic tendencies were reported by 35.1% of the models versus 20.2% of controls.

**Conclusion:**

Fashion models are at risk for the development of eating disorders. Even though not yet included in the DSM-5, the assessment of orthorexia nervosa among fashion models seems to be important. It is suggested to take appropriate measures to prevent the spread of disordered eating habits among models as they can lead to the development of anorexia nervosa or bulimia nervosa.

**Level of evidence:**

Level III, well-designed cohort study.

## Introduction and aims

Orthorexia nervosa (ON), an obsession with healthy eating, not yet included in the DSM-5 TR, shows similarities with obsessive–compulsive disorder (OCD) [1], yet it is more associated to eating disorders (EDs) than to OCD [2]. ON was first described by Bratman [3] as an obsessive, oftentimes extreme, and physically damaging disorder, related to but different from anorexia nervosa (AN), it is suggested that ON may represent a subtype of AN [4]. ON is characterised by the consumption of food considered to be pure and healthy, spending excessive time on purchasing the right ingredients and on preparing the appropriate meal often leading to a restrictive diet and social isolation. There are two different perspectives regarding the relationship between ON and AN. Some authors consider ON to be a subcomponent of AN, while others view ON as a distinct ED. In a systematic review, symptoms were found to be related to restraint and weight loss efforts, but not to body dissatisfaction or dysregulated eating. Therefore, the authors suggest that ON may be a distinct ED [5]. On the other side, it was also found that orthorexic eating behaviours might serve as a coping strategy in anorexic individuals [6].

Not only the desire to be healthy effects one’s food choice, but sociocultural influences have proven to have a strong negative effect on body image and eating habits [7]. Certain appearance-related professions that require a low body weight (e.g., ballet dancers, marathon runners, flight attendants) experience a higher risk for the development of EDs [8]. Professional fashion models are also at higher risk for the development of EDs due to the intense pressure in the fashion industry to maintain a thin body frame [9]. Fashion models are encouraged to use body size modifying methods to meet the industry’s requirements [10]. The frequency of ON is higher among performance artists [11] and participation in the fitness industry is also linked to higher ON tendencies [12, 13].

Modelling is a competitive milieu [14], which is overflooded with very young and very thin girls [15]. Social comparison is a normal behaviour strategy to better understand one’s position compared to other people [16]. Fashion models often must wait together at castings where they have the possibility to compare themselves to each other [17]. This comparison relates to physical appearance and dieting habits [9]. Such events could exacerbate social comparison between models. Not only models compare themselves to each other, but they are also compared to one another by other industry professionals (e.g., agents, casting directors, designers). These comparisons are almost solely based on measurements (thinness) and facial features of the models [18]. Social influence, especially on the appearance of one’s body is a risk factor for ED [19].

A form of dieting, called “clean eating” has gained wide popularity in the past years [20]. Clean eating refers to a diet pattern of avoiding food with artificial ingredients, high sugar and salt content and that favours foods from natural sources [21]. Due to the social appraisal of such form of eating, ED symptoms can remain hidden, as orthorexic behaviour could be a socially accepted form of AN tendencies [22]. The relationship between ON and other EDs is complex because ON may be a risk factor for EDs, and vice versa. For instance, ON could precede or follow AN [23].

Before an important job, models usually use dieting methods to “detoxify” their body which are oftentimes encouraged by their agents [10]. These methods may seem healthy at first, but the restrictive nature of such diets potentially have health damaging effects [24]. Fashion models are a high-risk group for osteoporosis, amenorrhoea, or cardiac complications due to malnutrition [25].

ON is characterised with intense control towards food, similarly to AN [21]. The sense of exerting control and making autonomous decisions can be an important factor in the development of AN [26]. Models face a particularly uncertain work schedule [27]. This can create the feeling of lack of control, which potentially leads to the necessity to gain control over their food intake.

Fashion models show higher risk for the development of subclinical AN than the general population [28]. This could be explained by fashion models being relatively young, they mainly engage in physical activity to control their body size and they face lack of control in their professional career [18]. The increased risk for classic EDs might enhance the frequency of orthorexic behaviours as well. To the best of our knowledge, no data has been published regarding the frequency of ON among fashion models. The present study is an exploratory research aiming to fill this gap and to shed some light on whether fashion models are more at risk to develop ON than non-models.

## Methods

### Design

The present manuscript is related to a larger epidemiological research conducted among models [28]. One independent part of this study is the examination of ON. An online screening questionnaire was administered, including demographic (i.e. age, sex, nationality, race, marital status) and anthropometric data (i.e. height, weight, BMI), the Eating Behaviour Severity Scale (EBSS), the Eating Disorder Inventory (EDI), and the 18-item Eating Habits Questionnaire.

### Recruitment

Snowball sampling was used because no reliable sampling frame was available to reach the model population. The questionnaire was circulated by personal contacts (N.B. has worked in the fashion industry as a model for five years), by social media platforms, by non-profit organisations concerned with the health status and interest of models (Humans of Fashion, Model Law, Models Empowered, The Models’ Health Pledge, Responsible Models Trust).

The non-model control group was recruited by snowball sampling as well, mostly from several international universities. Control group was adjusted to age to ensure same selection between models and non-models.

Informed consent was obtained from all individual participants included in this study. Information about the research was provided at the beginning of the online questionnaire where participants’ consent was required to continue responding.

### Participants

The participants were international fashion models and non-models, as a control group. 196 models and 305 non-models filled the questionnaire. The following inclusion criteria were used in the model group: females with at least one year in a model career, 16–37 years age limit, height ≥ 170 cm and BMI ≤ 25. No ‘plus size’ models were included, as the aim of the study was to assess straight size high fashion models, and there is a mutual relationship between obesity and certain psychopathological conditions, like higher body dissatisfaction and a tendency to strongly internalise the thin ideal, or depression [29]. In case of the control group, similar age (16–37 years) limits were used, and only female respondent were included. No further inclusion or exclusion criteria were used. Exclusion for BMI among the controls were not used as an inclination towards healthy eating obsession may be prevalent among individuals with muscular body composition, which can result in a higher BMI [30], the phenomena very unlikely in models, as muscular body types are very unfavored in the high fashion realm [9].

The subjects who provided incomplete data regarding age, height or weight were omitted from the analysis. The survey was kept confidential and anonymous, and participation was voluntary.

The research is in accordance with the Helsinki Declaration and was approved by the Regional Research Ethical Board of the Semmelweis University Budapest (No. 3/2020).

### Measures

The online survey used the English version of the EHQ that is a widely used measurement tool for the assessment of ON, with good psychometric properties [31]. The EHQ assesses cognitive, behavioural and emotional aspects of ON on three subscales. The Knowledge subscale measures the respondent’s knowledge of healthy eating, the Problems subscale assesses the problems associated with healthy eating and the Feelings subscale measures positive feelings about healthy eating. Due to the different language background (for most of the respondents English is their second language) and the previous uncertainties in the factorial structure of EHQ [e.g., 32–34], the questionnaire was fully re-evaluated on the same sample which lead to a 18-item version of the questionnaire with three similar subscales (Knowledge, Problems, Feelings). During the re-evaluation, confirmative factor analysis underpinned factorial validity, convergent validity against EDI was proved by correlation, reliability was supported by Cronbach’s alpha. The full re-evaluation paper is currently under peer-review, but available as preprint [35]. All three subscales were used in this present study and the internal consistencies were appropriate in both the model and control subsamples: in the Knowledge subscale, Cronbach’s alpha was 0.81/0.79 (model/control subsamples); in the Problems subscale 0.91/0.87; and in the Feelings subscale 0.80/0.77. Respondents who were in the top 25 percentile of the total EHQ score and whose average Likert ratings for both EHQ-P and EHQ-F were at least 2 were classified as having an ON tendency [36].

The Knowledge subscale was filled in by 171 models and 252 controls, the Problems subscale was filled in by 170 models and 252 controls and the Feelings subscale was filled in by 171 models and 255 controls. Incomplete subscales were not used in the analysis.

### Statistical analysis

In the statistical analysis the SPSS 23 software was administered. In the descriptive statistics averages and standard deviations were calculated. In the comparison of group means the *t*-tests were used because of the large sample size and Cohen’s *d* was used to determine the effect size. The Chi-square test was applied to compare proportions and Cramer’s *V* was used to assess effect size.

## Results

The sample consisted of 179 models and 261 non-models. The mean age of the two groups was similar: 25.9 ± 4.7 years and 25.0 ± 4.97 years, respectively. The age range was 16–37 years in both groups. The models were significantly taller: the mean height was 177.3 ± 3.58 cm vs. 167.4 ± 6.59 cm, *p* < 0.001. The range was 170.0–188.0 cm, and 150.0–188.0 cm, respectively. The mean BMI of the models was 18.1 ± 1.68, while for controls it was 22.1 ± 4.23 (*p* < 0.001).

Forty-four percent of the models showed BMI values between 17.0–18.5, and 12.3% of the controls. Twenty-one percent of the models, and 4.2% of the control subjects were severely underweight (BMI < 17.0).

The self-reported ethnic distribution of the models and controls was diverse: 56.4% vs. 92.3% white, 2.8% vs. 2.7% Asian, 3.4% vs. 1.5% black, 7.8% vs. 3.4% other. The proportion of missing data relating to the ethnicity was 29.5% among models, but all the control subjects responded. The participants represented 36 countries including but not limited to Austria, Belgium, Botswana, Canada, Ecuador, France, Iran, Korea, Norway, Pakistan, Russia, Tonga, and Vietnam. Most participants were from Hungary, France, Russia, the Netherlands, and the United States of America.

Regarding the results of the EHQ, models showed significantly higher scores on all three subscales compared to non-models. (Table 1).

**Table 1:** Comparison of EHQ subscales between fashion models and controls

	Fashion models			Controls			*t*	*p*	Cohen’s *d*
	*N*	*M*	SD	*N*	*M*	SD			
EHQ-K	171	2.42	0.70	252	2.08	0.63	5.203	< 0.001	0.52
EHQ-P	170	1.93	0.73	252	1.61	0.59	4.727	< 0.001	0.49
EHQ-F	171	3.20	0.61	255	2.96	0.65	3.830	< 0.001	0.38

*EHQ-K* Knowledge, *EHQ-P* Problems, *EHQ-F* Feelings

Regarding the proportion of those with higher average scores (≥ 2) on each subscale, we found that significantly more fashion models reached scores equal or over 2 on the Knowledge subscale compared to non-models, similarly on the Problems subscale the models showed significantly higher values. On the contrary, there was no significant difference between the two groups on the on the Feelings subscale of the EHQ, both groups reached high scores. Using the complex ON tendency assessment based on EHQ-P, EHQ-F and EHQ total score 35.1% of the models versus 20.2% of the control group considered to have ON tendencies (Table 2).

**Table 2:** Proportion of fashion models and controls ≥ 2 on mean of each subscale of EHQ and with ON tendency

	Fashion models		Controls		*Χ*^2^	*p*	Cramer’s *V*
	*N*	%	*N*	%			
EHQ-K ≥ 2	171	76.6	252	59.9	12.766	< 0.001	0.174
EHQ-P ≥ 2	170	44.1	252	25.0	16.859	< 0.001	0.200
EHQ-F ≥ 2	171	97.1	255	95.3	0.848	0.353	–
ON tendency	167	35.1	247	20.2	11.691	< 0.001	0.168

*EHQ-K* Knowledge, *EHQ-P* Problems, *EHQ-F* Feelings

## Discussion

This study is novel in assessing the risk of ON among fashion models. In this study, it was found that fashion models are a high-risk group for the development of ON. ON is a controversial concept and has not been recognised as a psychiatric disorder in the DSM-5 TR, consensus is pending on its classification. ON, often viewed as a socially approved form of eating concerns due to its association with higher moral standards and elitism [1], may hinder its recognition. This perception could obscure its potential progression into more severe EDs as the symptoms progress [23].

The frequency of subclinical AN is significantly higher among models (14.6%) than in control subjects (2.7% [28]). The tendency of ON seems even higher. In the present study 44.1% of models reached high scores on the Problems subscale of the EHQ (EHQ-P) compared to 25.0% of control group. On the other hand, in the proportion of respondent with higher score on the Feelings subscale (EHQ-F), there was no significant difference between the two groups. These results could be interpreted by the assumption that EHQ-F measures healthy orthorexia (HeOr), while EHQ-P the pathological dimensions [35]. HeOr is an increased interest in diet, and healthy eating behaviour as one’s identity [37]. The high score of EHQ-F in the control group could indicate that preoccupation with healthy food might be normative in today’s society just as the internalisation of the slim beauty ideal [38]. These are not signs of psychopathology, however, raises the awareness for the risk of developing ON. The ratio of pathological preoccupation with healthy eating, as indicated by high scores on the EHQ-P and tendencies towards ON based on a detailed EHQ assessment, is significantly higher among fashion models compared to non-models. A possible reason behind ON being overseen in the fashion industry is because modelling agents usually promote clean or pure diets to the models [10]. It seems more ethical to convince young girls to eat sugar-free, low carb, organic, unprocessed food from natural sources so that they would become healthier, and at the same time it is easier to maintain their measurements. These reasonings seem gentler than explicitly asking a young model to go on restrictive diets to lose weight. More so, because such clean and pure diets are already restrictive enough to cause malnutrition and thus, weight loss [39]. This diet and its result in a thin body frame can be then easily justified as simply eating pure and avoiding processed food in order to reach one’s maximum health which in reality is a disordered eating habit in development [40]. Modelling agents also advise models to do certain physical activities in order to tone up or to shape their bodies [10]. Certain sports, such as fitness and yoga has been associated with higher ON prevalence [12, 41]. Also, it is important to mention, that modelling holds very uncertain working conditions [27]. The lack of control over one’s own career and success can be upsetting [18]. Restricting the quality (and quantity) of food one eats can give a sense of control [21]. Such obsession around eating can result in the development of AN or BN [42]. The restrictive diets, especially at younger age (fashion models usually start their career as minors) can cause serious health implications. Hair loss, amenorrhoea, cardiac complications, osteoporosis are all serious consequences of an imbalanced diet and fashion models are at risk of such complications [25]. Clean eating is normalised in our culture; thus, such disordered eating habits remain hidden [40].

Further reason for the higher risk of ON among models may include engaged social media usage. There has been a link found between social media usage and ON tendencies [43, 44]. The number of Instagram followers became an important criterion at castings for choosing the right model for a job as clients believe they can reach a wider audience on the personal profiles of the models [9]. However, increased Instagram use is linked with higher ON tendencies [45]. The use of social media also negatively correlates with body image [7]. These findings can further point out the importance of the awareness towards EDs among fashion models. Social media is not only an important platform for social comparison regarding one’s appearance [12], but Instagram was also found to play an important role in nutrition-related decision-making [46]. This can influence both fashion models and the general population [47].

Higher income level can also be a factor why ON is more frequent among fashion models. A study found a correlation between income levels and ON, revealing a 68.5% tendency for orthorexia in high-income families [44]. Modelling is regarded as a lucrative occupation [17]. Eating healthy is fashionable [20] and those who do not keep an eye on their eating habits are usually negatively portrayed [48]. In a qualitative study both upward and downward comparison has been proven to play a role in the social aspect of the development of ON and potentially other EDs [40]. Eliminating certain food groups from one’s diet and following strict food rules gives a sense of superiority of self towards less healthy individuals, and an urge to resemble to those perceived as healthier is the basis of upward comparison. The impact of food prices on consumption can be an important factor as well, as the price of healthy food is higher, meaning that higher-income groups have easier access to them [49].

### Strength and limitations

To the best of our knowledge, this is the first publication on the risk of ON among fashion models. A thorough line of anthropometric questions and a validated questionnaire (EHQ) was used in this research to assess the risk of ON among fashion models. This study based on an internationally heterogenous sample may serve as a pioneer in the research of the pathogenetical background of ON. The study holds public health implications and raises awareness of fashion models being a high-risk group for the development of EDs.

Certain limitations have to be considered regarding this study. ON has no exact diagnostic criteria yet and it is still not a distinct nosological entity and not included in the DSM-5-TR. Participants received the survey during the COVID-19 pandemic when less modelling jobs were available. Models were potentially under less pressure to monitor their eating habits. Moreover, the self-reported anthropometric data may differ from the actual values, so the calculation of BMI may be distorted [50]. Socioeconomic and cultural background comparison were not conducted between the study group and the control group.

Further limitation of our research is that only female models were included in the research, no ‘plus size’ models were recruited, while the control group was selected from university students. It should be also considered that the distribution of BMI among control group compared to model group is significantly higher. However, it can be seen as part of the phenomenon that was investigated as orthorexia is not independent from BMI. Varga et al. [51] explain that in case of ON the inappropriate diet might cause weight loss.

It is also noticeable that the control group shows rather high scores on certain subscales. This can be reasoned with the fact that the majority of the control group participants are university students who are also a high-risk group regarding the development of EDs [52, 53]. This comparison makes our findings about fashion models even more alarming, as they reached higher scores on the EHQ compared to an already high scoring population.

### What is already known on this subject?

The frequency of subclinical AN is significantly higher among models (14.6%) than in control subjects (2.7% [28]). It was found that performance artists portray higher ON prevalence [11]. However, no data has been published on ON tendencies amongst models.

### What this study adds?

The current research showcases the increased frequency of ON among models (35.1% versus 20.1% of control group). This study further highlights the importance of preventive actions for the purpose of decreasing the risk factors for the development of EDs among fashion models.

### Future directions

Longitudinal studies would be favoured in order to better understand the risk factors and the prevalence of EDs among fashion models. Such studies are crucial to protect models from the manifestation of EDs. By protecting fashion models, media consumers would also be under less pressure to conform to extremely thin beauty ideals and their restrictive diets. The responsibility of the representatives of fashion industry has to be stressed to secure the fashion models' physical and mental wellbeing. The introduction of health protection regulation should be of key importance.

## Conclusion

Fashion models are at a higher risk for the development of ON than non-models due to the intense pressure to maintain specific size requirements enforced by the fashion industry. Orthorexic tendencies can lead to the manifestation of more severe EDs such as AN or BN. Implementing prevention strategies and strict regulations would be of great importance to avoid the development of EDs among fashion models and those young individuals most exposed to images representing such thin beauty ideals.

## Data Availability

The datasets generated during and/or analysed during the current study are available from the corresponding author on reasonable request.

## Abbreviations

AN: Anorexia nervosa
BN: Bulimia nervosa
DSM-5 TR: Diagnostic and Statistical Manual of Mental Disorders, Fifth Edition, Text Revision
EBSS: Eating Behaviour Severity Scale
EDI: Eating Disorder Inventory
HeOr: Healthy orthorexia
OCD: Obsessive–compulsive disorder
ON: Orthorexia nervosa
EHQ: Eating Habits Questionnaire
EHQ-F: Feeling subscale of Eating Habits Questionnaire
EHQ-K: Knowledge subscale of Eating Habits Questionnaire
EHQ-P: Problems subscale of Eating Habits Questionnaire

## References

[1] Mathieu J (2005). What is orthorexia?. J Am Diet Assoc.

[2] Zagaria A, Vacca M, Cerolini S, Ballesio A, Lombardo C (2022). Associations between orthorexia, disordered eating, and obsessive–compulsive symptoms: a systematic review and meta-analysis. Int J Eat Disord.

[3] Bratman S (1997) Orthorexia nervosa. Yoga Journal, October. https://www.orthorexia.com/original-orthorexia-essay/. Accessed 15 Oct 2015

[4] Cosh SM, Olson J, Tully PJ (2023). Exploration of orthorexia nervosa and diagnostic overlap with eating disorders, anorexia nervosa and obsessive-compulsive disorder. Int J Eat Disord.

[5] Atchison AE, Zickgraf HF (2022). Orthorexia nervosa and eating disorder behaviors: a systematic review of the literature. Appetite.

[6] Barthels F, Meyer F, Huber T, Pietrowsky R (2017). Orthorexic eating behaviour as a coping strategy in patients with anorexia nervosa. Eat Weight Disord.

[7] Aparicio-Martinez P, Perea-Moreno AJ, Martinez-Jimenez MP, Redel-Macías MD, Pagliari C, Vaquero-Abellan M (2019). Social media, thin-ideal, body dissatisfaction and disordered eating attitudes: an exploratory analysis. Int J Environ Res Public Health.

[8] Abraham S (1996). Eating and weight controlling behaviours of young ballet dancers. Psychopathol.

[9] Bogár N, Túry F (2019). The fashion industry and eating disorders: the dangers of the catwalk.

[10] Rodgers RF, Ziff S, Lowy AS, Yu K, Austin SB (2017). Results of a strategic science study to inform policies targeting extreme thinness standards in the fashion industry. Int J Eat Disord.

[11] Aksoydan E, Camci N (2009). Prevalence of orthorexia nervosa among Turkish performance artists. Eat Weight Disord.

[12] Eriksson L, Baigi A, Marklund B, Lindgren EC (2008). Social physique anxiety and sociocultural attitudes toward appearance impact on orthorexia test in fitness participants. Scand J Med Sci Sports.

[13] Bóna E, Szél Z, Kiss D, Gyarmathy A (2019). An unhealthy health behavior: analysis of orthorexic tendencies among Hungarian gym attendees. Eat Weight Disord.

[14] Johnson K (2011). Importing the flawless girl. Nev L J.

[15] Scully J (2016) How fashion became about power, and lost its ability to dream. Business of Fashion VOICES. https://www.youtube.com/watch?v=5L5uYojEJTw

[16] Mills JS, Shannon A, Hogue J, Levin MP (2017). Beauty, body image, and the media. Perception of beauty.

[17] Mears A (2011). Pricing beauty. The making of a fashion model.

[18] Dauxerre V (2017). Size zero: my life as a disappearing model.

[19] Corning AF, Krumm AJ, Smitham LA (2006). Differential social comparison processes in women with and without eating disorder symptoms. J Couns Psychol.

[20] Nevin SM, Vartanian LR (2017). The stigma of clean dieting and orthorexia nervosa. J Eat Disord.

[21] Koven NS, Abry AW (2015). The clinical basis of orthorexia nervosa: emerging perspectives. Neuropsychiatr Dis Treat.

[22] Cartwright MM (2004). Eating disorder emergencies: understanding the medical complexities of the hospitalized eating disordered patient. Crit Care Nurs Clin North Am.

[23] Brytek-Matera A, Rogoza R, Gramaglia C, Zeppegno P (2015). Predictors of orthorexic behaviours in patients with eating disorders: a preliminary study. BMC Psychiatry.

[24] Musolino C, Warin M, Wade T, Gilchrist P (2015). “Healthy anorexia”: the complexity of care in disordered eating. Soc Sci Med.

[25] Bogár N, Túry F, Pászthy B (2022). Risks of health deterioration in the fashion industry [Hungarian]. Orvosképzés.

[26] Tan JOA, Hope T, Stewart A, Fitzpatrick R (2003). Control and compulsory treatment in anorexia nervosa: the views of patients and parents. Int J Law Psychiatry.

[27] Bogár N, Pászthy B, Túry F (2021). Health protective regulation of the fashion industry [Hungarian]. Hungarian Med J.

[28] Bogár N, Dukay-Szabó S, Simon D, Túry F, Pászthy B (2022). Frequency of disordered eating habits among fashion models. Eur Eat Disord Rev.

[29] Rajan TM, Menon V (2017). Psychiatric disorders and obesity: a review of association studies. J Postgrad Med.

[30] Oberle CD, Lipschuetz SL (2018). Orthorexia symptoms correlate with perceived muscularity and body fat, not BMI. Eat Weight Disord.

[31] Gleaves DH, Graham EC, Ambwani S (2013). Measuring “orthorexia”: development of the Eating Habits Questionnaire. Int J Educ Psychol Assess.

[32] Godefroy V, Trinchera L, Dorard G (2021). Optimizing the empirical assessment of orthorexia nervosa through EHQ and clarifying its relationship with BMI. Eat Weight Disord.

[33] Brytek-Matera A, Plasonja N, Décamps G (2020). Assessing orthorexia nervosa: validation of the Polish version of the Eating Habits Questionnaire in a general population sample. Nutrients.

[34] Halim ZM, Dickinson KM, Kemps E, Prichard I (2020). Orthorexia nervosa: examining the Eating Habits Questionnaire’s reliability and validity, and its links to dietary adequacy among adult women. Public Health Nutr.

[35] Simon D, Bogár N, Dukay-Szabó S, Túry F (2023). Re-evaluation and revision of Eating Habits Questionnaire. MedRxiv.

[36] Hayatbini N, Oberle CD (2019). Are orthorexia nervosa symptoms associated with cognitive inflexibility?. Psychiatry Res.

[37] Depa J, Barrada JR, Roncero M (2019). Are the motives for food choices different in orthorexia nervosa and healthy orthorexia?. Nutrients.

[38] Homan K (2010). Athletic-ideal and thin-ideal internalization as prospective predictors of body dissatisfaction, dieting, and compulsive exercise. Body Image.

[39] Moroze RM, Dunn TM, Craig Holland J, Yager J, Weintraub P (2015). Microthinking about micronutrients: a case of transition from obsessions about healthy eating to near-fatal “orthorexia nervosa” and proposed diagnostic criteria. Psychosom.

[40] Greville-Harris M, Smithson J, Karl A (2019). What are people’s experiences of orthorexia nervosa? A qualitative study of online blogs. Eat Weight Disord.

[41] Bartrina JA (2007) Ortorexia o la obsesión por la dieta saludable. Arch Latinoam Nutr 57:313–315. http://ve.scielo.org/scielo.php?script=sci_arttext&pid=S0004-06222007000400002&lng=es&tlng=es. 18524314

[42] Brytek-Matera A, Donini LM, Krupa M, Poggiogalle E, Hay P (2015). Orthorexia nervosa and self-attitudinal aspects of body image in female and male university students. J Eat Disord.

[43] Cohen R, Blaszczynski A (2015). Comparative effects of Facebook and conventional media on body image dissatisfaction. J Eat Disord.

[44] Yilmazel G (2021). Orthorexia tendency and social media addiction among candidate doctors and nurses. Perspect Psychiatr Care.

[45] Turner PG, Lefevre CE (2017). Instagram use is linked to increased symptoms of orthorexia nervosa. Eat Weight Disord.

[46] Mete R, Shield A, Murray K, Bacon R, Kellett (2019). What is healthy eating? A qualitative exploration. Public Health Nutr.

[47] Klassen KM, Douglass CH, Brennan L, Truby H, Lim MSC (2018). Social media use for nutrition outcomes in young adults: a mixed-methods systematic review. Int J Behav Nutr Phys Act.

[48] Puhl RM, Peterson JL, DePierre JA, Luedicke J (2013). Headless, hungry, and unhealthy: a video content analysis of obese persons portrayed in online news. J Health Commun.

[49] Andreyeva T, Long MW, Brownell KD (2010). The impact of food prices on consumption: a systematic review of research on the price elasticity of demand for food. Am J Public Health.

[50] Giacchi M, Mattei R, Rossi S (1998). Correction of the self-reported BMI in a teenage population. Int J Obes.

[51] Varga M, Dukay-Szabó S, Túry F (2013). Orthorexia nervosa and its background factors [Hungarian]. Ideggyogy Sz.

[52] Parra-Fernández ML, Rodríguez-Cano T, Onieva-Zafra MD, Perez-Haro MJ, Casero-Alonso V, Fernández-Martinez E (2018). Prevalence of orthorexia nervosa in university students and its relationship with psychopathological aspects of eating behaviour disorders. BMC Psychiatry.

[53] Erol Ö, Özer A (2019). Determination of orthorexia nervosa symptoms and eating attitudes in medicine students. Eur J Public Health.

